# New Approach for the One-Pot Synthesis of 1,3,5-Triazine Derivatives: Application of Cu(I) Supported on a Weakly Acidic Cation-Exchanger Resin in a Comparative Study

**DOI:** 10.3390/molecules24193586

**Published:** 2019-10-05

**Authors:** Eva Havránková, Jozef Csöllei, Pavel Pazdera

**Affiliations:** 1 Department of Chemical Drugs, Faculty of Pharmacy, University of Veterinary and Pharmaceutical Sciences, Brno 612 42, Czech Republic; Csolleij@vfu.cz; 2 Department of Chemistry, Faculty of Science, Centre for Syntheses at Sustainable Conditions and Their Management, Masaryk University, Brno 625 00, Czech Republic; pazdera@chemi.muni.cz

**Keywords:** 1,3,5-triazine, supported Cu(I) catalyst, Ullmann reaction, one-pot synthesis

## Abstract

An efficient and simple methodology for Ullmann Cu(I)-catalyzed synthesis of di- and trisubstituted 1,3,5-triazine derivatives from dichlorotriazinyl benzenesulfonamide and corresponding nucleophiles is reported. Cations Cu(I) supported on macroporous and weakly acidic, low-cost industrial resin of polyacrylate type were used as a catalyst. The reaction times and yields were compared with traditional synthetic methods for synthesis of substituted 1,3,5-triazine derivatives via nucleophilic substitution of chlorine atoms in dichlorotriazinyl benzenesulfonamide. It was found that Ullmann-type reactions provide significantly shortened reaction times and, in some cases, also higher yields. Finally, trisubstituted *s*-triazine derivatives were effectively prepared via Ullmann-type reaction in a one-pot synthetic design. Six new *s*-triazine derivatives with potential biological activity were prepared and characterized.

## 1. Introduction

**Figure 1:**
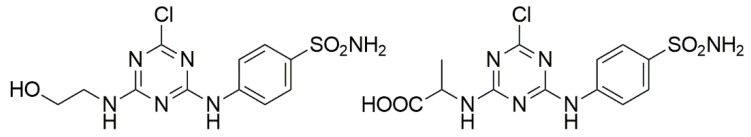
Example of a carbonic anhydrase inhibitors structure [10].

The substituted 1,3,5-triazine derivatives show a wide spectrum of effects on biological systems such as anti-bacterial, anti-viral, anti-mycobacterial, anti-tubercular, anti-malarial, anti-leishmanial, anti-amoebic, anti-inflammatory, anti-HIV, anti-cancer, and others [1,2,3,4]. A variety of biological activities of triazine derivatives has attracted attention in the field of medicinal chemistry research. Especially, triazinylaminoalkylbenzenesulfonamides are intensively studied because of their antimicrobial and anticancer activity [5,6,7,8,9]. They act as inhibitors of carbonic anhydrase and the key structural moiety for biological activity in these derivatives is aminoalkylbenzenesulfonamide (examples of the inhibitors are given in Figure 1).

Substituted 1,3,5-triazines are usually synthesized in two different ways. The classic method is based on the nucleophilic substitution of chlorine atoms in starting 2,4,6-trichloro-1,3,5-triazine, i.e., cyanuric chloride [11,12,13,14]. Another approach to synthesis is a sequential construction of a triazine ring [15,16,17]. Zhang et al. [18] recently published a copper-catalyzed synthesis (construction of a triazine ring) of substituted 2,4-diamino-1,3,5-triazines from 1,1-dibromoalkenes and biguanides under mild conditions.

Copper based catalytic systems used for Ullmann C–N coupling are in most cases used under conditions of a homogenous catalysis and in the presence of ligands, which are necessary for stabilization of Cu(I)- and Cu(III)-intermediates [19,20]. In last twenty years, only few heterogeneous/solid supported reusable Cu-catalysts suitable for Ullmann coupling reactions were developed. Interesting examples of the abovementioned exceptions were cellulose-supported copper(0) [21], an iminopyridine ligand grafted onto phosphorus dendrimers [22], supported copper fluorapatite [23,24], copper nanoparticles [25], Cu_2_O-coated Cu-nanoparticles [26], titanium oxide encapsulated copper nanocatalyst [27], furfural functionalized polymer-amine grafted with copper catalyst [28], *N*-heterocyclic carbene–copper complex supported on hyper crosslinked polymers [29], and glass beads coated with Cu/Zn nanocatalyst [30] or silica-tethered copper complex [31].

Solid supported catalysts combine advantages of homogenous and heterogeneous catalysts [32,33,34]. Commercially available industrial cation exchange resins, which are very cheap and easily available, proved to be an optimal supporting material for this type of heterogeneous catalyst [34,35,36,37,38]. Modified cation exchange resins supporting metal ions as catalytic systems have many advantages (i.e., they are very effective, easily separable and stable).

In this paper, we report an efficient and simple synthetic approach for preparation of 1,3,5-triazine derivatives carried out as a one-pot reaction and catalyzed by Cu(I)-supported on a weekly acidic resin.

## 2. Results and Discussion

Our heterogeneous catalyst containing Cu(I)-cations supported on a cation exchange resin is, in comparison with most of the previously reported heterogeneous Cu(I) catalysts, very easy for preparation and very cheap. Large size of resin beads allows the separation of catalyst more easily than other solid supports (i.e., alumina, silica, carbon, zeolite). Also, unlike the other hard supports, the presented catalytic system does not rub against the surface of reaction vessels. Because of these reasons, the catalyst can be easily used in industrial production. The recoverability of catalyst was also studied (see Supporting Information). It was found, that catalyst can be used in one-pot synthesis for ten times without loss of a catalytic activity.

**Scheme 1:**
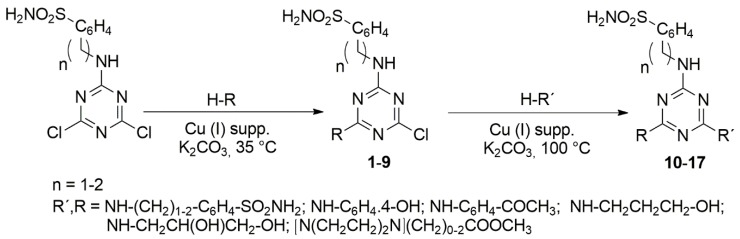
General scheme of syntheses of di- and trisubstituted 1,3,5-triazine derivatives.

The catalytic efficiency of Cu(I)-cations supported on a macroporous weakly acidic cation exchanger in syntheses of di- and trisubstituted 1,3,5-triazine derivatives starting from dichlorotriazinyl benzenesulfonamide and corresponding nucleophiles was studied in this research. The catalyzed reactions were performed (see Section 3) with the addition of 2.5 mol% of Cu(I)-supported on a given resin. The general scheme of the reactions was demonstrated by Scheme 1. The resulting reaction times and obtained yields of catalyzed reactions are summarized in Table 1 together with the results of uncatalyzed reactions of the same type (the %yields of reactions and spectral characterization of compounds were published recently in Reference [39]).

It is well known that the best results for Ullmann-type synthesis catalyzed by Cu(I) are achieved under specific conditions as there are many variables [40]. Potassium carbonate (inorganic base) is recommended as one of the most effective bases for Ullmann couplings especially together with *N,N*-dimethylformamide as a solvent. These findings were also confirmed by the obtained results.

Data in Table 1 clearly shows that employment of Cu(I) supported catalytic system lead to significantly shorter reaction times and usually provided better yields than those observed in case of uncatalyzed reactions. 

The side products were not observed even in reactions with strong nucleophiles. The first reason is that aminoalkylbenzenesulfonamide structural motive present in starting compound rapidly decreases the reactivity of remaining chlorine atoms on triazine skeleton. It was also observed, that occurrence of side products can be well controlled by the reaction temperature. In the reactions with lower yields, the conversion of starting nucleophile was not full, and the reaction was terminated when the rate of conversion did not change after 24 h.

The general rule of nucleophilicity applies—the better the nucleophile, the better the results in reaction time. Therefore, in the synthesis of compounds **1**, **3**, and **5** the reaction time is shortened with the increasing number of CH_2_ groups between the piperazine ring and the terminal methoxycarbonyl (EWG) which improves the nucleophilicity of the second piperazine nitrogen atom.

On the other hand, Table 1 shows the reversed effect of the present sulfonamide group (EWG) on the reactivity of the triazine ring when substituting the chlorine atom. The reactivity of the triazine skeleton decreases with the increasing distance of the sulfonamide group, i.e., with the increasing number of CH_2_ groups. Surprisingly, for compounds containing aminopropanole substituent, the opposite trend was observed.

As an additional contribution of our study, a simplified design of the synthesis was proposed and successfully verified. The catalyzed syntheses of trisubstituted derivatives of *s*-triazine (compounds **10**–**17**) can now be carried out as one-pot reactions which brings further benefits. The reaction times of one-pot syntheses are comparable with those of step-by-step substitution reactions (see Table 1). The one-pot design of reactions moreover provides slightly higher yields.

The efficiency of our catalyst was also compared with CuI (see Table 2). Synthesis of compound **5** was carried out as described in experimental section (Section 3.3.1) and the supported catalyst was replaced with 2.5 mol% of CuI. We were pleased to find that use of CuI as a catalyst did not have any effect on the model reaction and the yield and reaction time were comparable with those of the uncatalyzed reaction (see Table 2). As an explanation of different catalytic activity of Cu(I)-cations supported on resin and free CuI, we supposed that the carboxylate groups present on the surface of resin could stabilize Cu(I) cations.

## 3. Experimental Section

### 3.1. General Information

All reagents were purchased from commercial suppliers (Sigma-Aldrich, Darmstadt, Germany) and used as supplied without further purification. Purolite C104 Plus (Purolite^®^ Worldwide, Bala Cynwyd, PA, USA) was used as a solid support. Characteristic properties of Purolite C104 Plus: weakly acidic polyacrylic cation-exchanger resin of macroporous type, H^+^ ionic form, total volume capacity 4.5 eq/L, specific gravity 1.19 g/mL (for further characteristics of the resin please see the Supplementary Information).

All the reactions were monitored by TLC performed on precoated Silica gel 60 F254 plates (Merck, Darmstadt, Germany). Methanol was used as eluent; UV light (254 and 356 nm) and ninhydrin reagent were used for detection of spots at 180 °C. NMR spectra were recorded on DRX 500 Avance (Bruker Biospin, Billerica, MA, USA) spectrometer using tetramethylsilane as an internal standard. The FTIR spectra were obtained on an Alpha II FTIR Spectrometer (Bruker, Billerica, MA, USA) equipped with ATR module. Melting points (uncorrected) were recorded on Kofler’s block Boetius Rapido PHMK 79/2106 (Wagetechnik, Dresden, Germany) with temperature gradient 4 °C.min^−1^.

All data of known compounds agreed with those of previously reported [39].

### 3.2. Catalyst Preparation and Characterization

Seventy-five grams of Purolite^®^ C104 Plus resin in Na^+^ form was stirred in 200 mL of water. Cupric acetate monohydrate (49.9 g, 0.25 mol) was dissolved in 750 mL of water and 85 mL of aqueous ammonia solution (commercially available 28 *w*/*w* % solution, 1.255 mol) was added. The resulting dark-blue solution was added to the resin suspension and stirred for 30 min. Then the aqueous phase was decanted and the blue solid washed with water until pH 7–8. Hydroxylammonium chloride (29.9 g, 0.43 mol) was dissolved in 250 mL of water and the resin was added into the solution and stirred at 50 °C until the color changed from blue to light gray (30 min). After the change of color, the solution was decanted and the solid was washed with water until neutral pH and then twice with methanol (300 mL).

The copper content determined by flame atomic absorption spectroscopy (Shimadzu, Kjóto, Japan) was approximately 0.078 mmol of Cu/1 g of a dry catalyst. The oxidation state of the copper present in the catalyst was confirmed by XPS (X-ray photoelectron spectroscopy, Kratos Analytical, Manchester, UK). The Cu 2p3/2 peak was located at 932.84 eV and the Cu 2p1/2 peak was located at 952.67 eV which is characteristic of the presence of Cu(I) in the catalyst [41]. The IR spectra of the catalyst in different stages of preparation are given in the Supplementary Materials. These spectra do not provide much information, but it confirms the presence of free hydroxy groups in the catalyst which helps to stabilize Cu(I)-cations.

### 3.3. General Synthetic Procedures

#### 3.3.1. General Method for Synthesis of Disubstituted Derivatives of Cyanuric Chloride as a Step-by-Step Reaction

Uncatalyzed reactions were performed according to the methodology published previously in [39].

Catalyzed reactions were performed according to the general method: Starting dichlorotriazinyl benzenesulfonamide (1 mmol; 0.320 g of 4-[(4,6-dichloro-1,3,5-triazin-2-yl)amino]-benzene-1-sulfonamide, 0.334 g of 4-{[(4,6-dichloro-1,3,5-triazin-2-yl)amino]methyl}-benzene-1-sulfonamide or 0.348 g of 4-{2-[(4,6-dichloro-1,3,5-triazin-2-yl)amino]ethyl}benzene-1-sulfonamide) was dissolved in 10 mL of DMF. Then 1 mmol (0.138 g) of solid anhydrous potassium carbonate was added in small portions and the mixture was stirred for 10 min. Then 1 mmol of the appropriate nucleophile was added portion wise. Finally, 2.5% mol of supported Cu(I) ions (312 mg of catalyst) were added into the reaction mixture. Reaction was stirred at 35 °C until the maximum conversion of starting reactants was achieved (monitored by TLC). After completion of a reaction, the catalyst and salt were filtered off. Crushed ice was then added into the solution and the formed precipitate was collected by filtration. The crude product was dissolved in hot acetone and precipitated by the addition of isopropyl alcohol.

#### 3.3.2. General Method for Synthesis of Trisubstituted Derivatives of Cyanuric Chloride as a Step-by-Step Reaction

Uncatalyzed reactions were performed according to the methodology published previously in [39].

Catalyzed reactions were performed according to the general method: Appropriate disubstituted *s*-triazine derivative (1 mmol) was dissolved in 10 mL of DMF. Then 1 mmol (0.138 g) of solid anhydrous potassium carbonate was added in small portions and the mixture was stirred for 10 min. Then 1 mmol of the appropriate nucleophile was added portion wise. Finally, 2.5% mol. of supported Cu(I) ions (312 mg of catalyst) was added into the reaction mixture. The reaction mixture was then stirred at 100 °C until the maximum conversion of starting nucleophile was achieved (monitored by TLC). After completion of the reaction, the catalyst and salt were filtered off. Crushed ice was then added into the solution and the formed precipitate was collected by filtration. The crude product was dissolved in hot acetone and precipitated by the addition of isopropyl alcohol.

#### 3.3.3. General Method for Synthesis of Trisubstituted Derivatives of Cyanuric Chloride as a One-Pot Reaction

Starting dichlorotriazinyl benzenesulfonamide (1 mmol; 0.320 g of 4- [(4,6-dichloro-1,3,5-triazin-2-yl)amino]benzene-1-sulfonamide, 0.334 g of 4-{[(4,6-dichloro-1,3,5-triazin-2-yl)amino]methyl}benzene-1-sulfonamide or 0.348 g of 4-{2-[(4,6-dichloro-1,3,5-triazin-2-yl)amino]-ethyl}benzene-1-sulfonamide)) was dissolved in 20 mL of DMF. Then 1 mmol (0.138 g) of solid anhydrous potassium carbonate was added in small portions and the mixture was stirred for 10 min. Then 1 mmol of the appropriate nucleophile was added portion wise. Finally, 2.5% mol. of supported Cu(I) ions (312 mg of catalyst) was added into the reaction mixture. Reaction was stirred at 35 °C until the maximum conversion of starting material is achieved (monitored by TLC). After completion of the first reaction step, 1 mmol of the second nucleophile and 1 mmol (0.138 g) of anhydrous potassium carbonate were added into the reaction mixture. The reaction mixture was then stirred at 100 °C until the maximum conversion of a nucleophile was determined (monitored by TLC). After completion of a reaction, the catalyst and salt were filtered off. Crushed ice was then added into the solution and the formed precipitate was collected by filtration. The crude product was dissolved in acetone and precipitated by the addition of isopropyl alcohol.

#### 3.3.4. Characterization of New Compounds

For % yields and reaction times see Table 1.

*4-[({4-Chloro-6-[(3-hydroxypropyl)amino]-1,3,5-triazin-2-yl}amino)methyl]benzenesulfonamide* (**6**). White solid; mp 212–214 °C. ^1^H-NMR (500 MHz, DMSO-*d*_6_) δ ppm 7.77 (2H, d, *J* = 8.1 Hz, CH), 7.48 (2H, d, *J* = 8.1 Hz, CH), 4.50 (2H, s, NH-CH_2_), 3.41–3.39 (2H, m, CH_2_-OH), 3.24–3.23 (2H, m, NH-CH_2_), 1.56–1.54 (2H, m, CH_2_). ^13^C-NMR (125 MHz, DMSO-*d*_6_) δ ppm 168.1, 165.9, 162.8, 144.8, 143.1, 127.8, 126.2, 58.9, 43.9, 38.1, 32.3. IR ν_max_ (cm^−1^) 3352 (OH, NH, NH_2_), 3243, 2942 (CH_arom_), 2918 (CH_alif_, CH_2alif_), 1602 (C=C_arom_), 1434, 1399, 1155 (SO_2_NH_2_).

*4-[2-({4-Chloro-6-[(3-hydroxypropyl)amino]-1,3,5-triazin-2-yl}amino)ethyl]benzenesulfonamide* (**7**). White solid; mp 228–229 °C. ^1^H-NMR (500 MHz, DMSO-*d*_6_) δ ppm 7.75 (2H, d, *J* = 8.1 Hz, CH), 7.40 (2H, d, *J* = 8.1 Hz, CH), 3.48–3.43 (2H, m, CH_2_-OH), 3.35–3.34 (2H, m, NH-CH), 3.27–3.25 (2H, m, NH-CH), 2.89–2.87 (2H, m, CH), 1.70–1.62 (2H, m, CH). ^13^C-NMR (125 MHz, DMSO-*d*_6_) δ ppm 168.6, 168.0, 165.8, 143.7, 143.1, 129.5, 126.3, 58.9, 42.1, 38.2, 34.9, 32.6. IR ν_max_ (cm^−1^) 3329 (NH, NH_2_), 3250, 2943 (CH_alif_, CH_2alif_), 1637 (C=C_arom_), 1447, 1405, 1154 (SO_2_NH_2_)

*4-[2-({4-Chloro-6-[(2,3-dihydroxypropyl)amino]-1,3,5-triazin-2-yl}amino)ethyl]benzenesulfonamide* (**8**). White solid; mp 185–186 °C. ^1^H-NMR (500 MHz, DMSO-*d*_6_) δ ppm 7.75 (2H, d, *J* = 8.1 Hz, CH), 7.42 (2H, d, *J* = 8.1 Hz, CH), 6.07 (6H, s, OH, NH, NH_2_), 3.89–3.85 (1H, m, CH-OH), 3.49–3.46 (4H, m, CH_2_), 3.37–3.32 (4H, m, CH_2_). ^13^C-NMR (125 MHz, DMSO-*d*_6_) δ ppm 168.5, 168.0, 165.7, 143.6, 143.2, 129.5, 126.1, 70.5, 64.5, 44.4, 42.0, 34.9. IR ν_max_ (cm^−1^) 3352 (OH, NH, NH_2_), 3221, 2984 (CH_Arom_) 2926 (CH_alif_, CH_2alif_), 2856, 1558 (C=C_Arom_), 1407 (SO_2_NH_2_), 1151, 1091 (C–OH).

*Methyl 3-(4-{4-Chloro-6-[(4-sulfamoylphenethyl) amino]-1,3,5-triazin-2-yl}piperazin-1-yl)propanoate* (**9**). Beige solid; mp 188–190 °C. ^1^H-NMR (500 MHz, DMSO-*d*_6_) δ ppm 7.81 (2H, d, *J* = 8.1 Hz, CH), 7.47 (2H, d, *J* = 8.1 Hz, CH), 3.97–3.87 (4H, m, CH_2_), 3.77–3.75 (2H, m, NH-CH_2_), 3.66 (3H, s, CH_3_), 2.98–2.96 (4H, m, CH_2_), 2.87–2.85 (2H, m, CH_2_), 2.59 (2H, t, *J* = 7.8 Hz, N-CH_2_), 2.12 (2H, t, *J* = 7.8 Hz, CH_2_-COO). ^13^C-NMR (125 MHz, DMSO-*d*_6_) δ ppm 172.8, 168.6, 165.9, 163.9, 143.9, 142.7, 129.6, 126.2, 53.5, 52.5, 51.8, 43.4, 41.9, 34.8, 31.9. IR ν_max_ (cm^−1^) 3323 (NH, NH_2_), 3242, 2952 (CH_arom_) 2913, 2844 (CH_alif_, CH_2alif_), 1683 (C=O), 1573 (C=C_arom_), 1360 (SO_2_NH_2_), 1331 (COC), 1142 (SO_2_NH_2_), 1081 (COC).

*Methyl 3-(4-{4-[(4-acetylphenyl)amino]-6-[(4-sulfamoylphenethyl)amino]-1,3,5-triazin-2-yl} piperazin-1-yl)propanoate* (**16**). Beige solid; mp 198–200 °C. ^1^H-NMR (500 MHz, DMSO-*d*_6_) δ ppm 8.87 (4H, br s, NH, NH_2_), 7.61 (2H, d, *J* = 8.0 Hz, CH), 7.53 (2H, d, *J* = 8.0 Hz, CH), 7.44 (2H, d, *J* = 7.8 Hz, CH), 7.36 (2H, d, *J* = 7.8 Hz, CH), 3.78–3.76 (4H, m, CH_2_), 3.63–3.59 (5H, m, CH_3_, NH-CH_2_CH_2_), 2.91–2.89 (2H, m, NH-CH_2_CH_2_), 282 (3H, s, CH_3_), 2.45–2.43 (4H, m, CH_2_), 2.38 (2H, t, *J* = 7.8 Hz, CH_2_CH_2_COO), 2.13 (2H, t, *J* = 7.8 Hz, CH_2_CH_2_COO). ^13^C-NMR (125 MHz, DMSO-*d*_6_) δ ppm 194.3, 172.9, 167.1, 166.8, 166.2, 146.3, 144.8, 142.3, 133.4, 129.6, 129.1, 126.2, 119.3, 51.7, 51.1, 49.9, 44.2, 43.6, 34.6, 31.9, 26.7. IR ν_max_ (cm^−1^) 3298, 3280 (NH, NH_2_), 3070, 3045, 3041 (CH_Arom_), 2974, 2949 (CH_alif_, CH_2 alif_), 1660 (C=O), 1631, 1597 (C=C, C=N), 1361 (SO_2_NH_2_), 1330 (COC), 1154 (SO_2_NH_2_), 1069 (COC)

*4-[2-({4-[(4-acetylphenyl)amino]-6-[(2,3-dihydroxypropyl)amino]-1,3,5-triazin-2-yl}amino)ethyl]benzenesulfonamide* (**17**). Beige solid; mp 210–213 °C. ^1^H-NMR (500 MHz, DMSO-*d*_6_) δ ppm 9.13 (5H, br s, NH, NH_2_), 7.9 (2H, d, *J* = 8.0 Hz, CH), 7.85 (2H, d, *J* = 8.0 Hz, CH), 7.73 (2H, d, *J* = 7.8 Hz, CH), 6.94 (2H, d, *J* = 7.8 Hz, CH), 6.57 (2H, br s, OH), 4.29–4.27 (1H, m, CH–OH), 3.61–3.54 (4H, m, NH-CH_2,_ NH-CH_2_), 2.92–2.90 (2H, m, NH-CH_2_CH_2_), 2.01 (3H, s, CH_3_). ^13^C-NMR (125 MHz, DMSO-*d*_6_) δ ppm 197.0, 166.9, 166.8, 165.8, 145.4, 144.2, 142.2, 132.1, 129.7, 129.6, 126.3, 116.4, 69.0, 62.1, 43.1, 42.9, 34.3, 26.3. IR ν_max_ (cm^−1^) 3352, 3316, 3067 (NH, NH_2_, OH), 2998, 2986, 2967 (CH_Arom_), 2954 (CH_alif_, CH_2 alif_), 1670 (C=O), 1635, 1584 (C=C, C=N), 1355 (SO_2_NH_2_), 1329 (COC), 1206 (C–OH), 1154 (SO_2_NH_2_), 1065 (COC).

## 4. Conclusions

The catalytic efficiency of Cu(I) cations supported on a macroporous weakly acidic cation exchanger in synthesis of 1,3,5-triazine derivatives was studied. The study brought two basic improvements into the synthesis of di- and trisubstituted triazine derivatives. Employment of supported Cu(I) catalyst lead to significantly shorter reaction times and, moreover, reactions usually provided better yields than uncatalyzed reactions. In the case of final trisubstituted *s*-triazine derivatives starting from monosubstituted triazine, a new one-pot synthetic design was successfully employed and verified bringing the additional benefit in higher yields and savings in labor and time. Six new *s*-triazine derivatives with potential biological activity were prepared and characterized using the new one-pot synthetic method.

## Figures and Tables

**Table 1:** Results of uncatalyzed and catalyzed syntheses of di- and trisubstituted 1,3,5-triazine derivatives including one-pot synthesis of trisubstituted 1,3,5-triazine derivatives.

Compound	n	R	R′	Without Catalyst		Catalyst-Supported Cu(I) ^b^		One-Pot Reaction; Catalyst-Supported Cu(I) ^b^	
				t (h)	%yield	t (h)	%yield	t (h)	%yield
**1 ^a^**	1	[N(CH_2_CH_2_)_2_N]COOCH_3_	Cl	168	63.4	10	67.2	-	-
**2 ^a^**	0	[N(CH_2_CH_2_)_2_N]COOCH_3_	Cl	64	89.3	4	87.9	-	-
**3 ^a^**	1	[N(CH_2_CH_2_)_2_N]CH_2_COOCH_3_	Cl	144	44.3	8	54.8	-	-
**4 ^a^**	0	[N(CH_2_CH_2_)_2_N]CH_2_COOCH_3_	Cl	50	86.9	3	88.1	-	-
**5 ^a^**	1	[N(CH_2_CH_2_)_2_N]CH_2_CH_2_COOCH_3_	Cl	72	29.8	2	59.9	-	-
**6**	1	NH-CH_2_CH_2_CH_2_OH	Cl	23	64.1	7	57.4	-	-
**7**	2	NH-CH_2_CH_2_CH_2_OH	Cl	27	97.9	3	95.2	-	-
**8**	2	NH-CH_2_CH(OH)CH_2_OH	Cl	13	87.6	5	77.2	-	-
**9**	2	[N(CH_2_CH_2_)_2_N]CH_2_CH_2_COOCH_3_	Cl	33	77.8	5	78.1	-	-
**10 ^a^**	1	NH-CH_2_CH_2_-C_6_H_4_(1,4)-4-SO_2_NH_2_	NH-CH_2_CH_2_-C_6_H_4_(1,4)-4-SO_2_NH_2_	22	32.0	2	51.9	12	56.9
**11 ^a^**	0	NH-CH_2_CH_2_-C_6_H_4_(1,4)-4-SO_2_NH_2_	NH-CH_2_CH_2_-C_6_H_4_(1,4)-4-SO_2_NH_2_	24	68.3	2	71.2	8	73.2
**12 ^a^**	1	NH-CH_2_-C_6_H_4_(1,4)-4-SO_2_NH_2_	NH-CH_2_CH(OH)CH_2_OH	30	45.5	4	62.9	16	67.1
**13 ^a^**	1	[N(CH_2_CH_2_)_2_N]COOCH_3_	NH-CH_2_CH(OH)CH_2_OH	72	20.2	8	46.0	18	51.4
**14 ^a^**	1	NH-C_6_H_4_(1,4)-4-OH	NH-CH_2_CH(OH)CH_2_OH	96	24.6	7	54.8	14	60.7
**15 ^a^**	1	[N(CH_2_CH_2_)_2_N]CH_2_COOCH_3_	NH-CH_2_CH(OH)CH_2_OH	72	27.0	5	53.9	13	56.8
**16**	2	[N(CH_2_CH_2_)_2_N]CH_2_CH_2_COOCH_3_	NH-C_6_H_4_(1,4)-4-COCH_3_	16	26.8	5	47.0	9	49.8
**17**	2	NH-CH_2_CH(OH)CH_2_OH	NH-C_6_H_4_(1,4)-4-COCH_3_	9	35.5	3	46.0	10	47.6

^a^ The spectral characteristics, yields of non-catalyzed reactions, and general methodology for non-catalyzed synthesis of these compounds were previously reported in Reference [39]. ^b^ Detailed reaction conditions are reported in the experimental section.

**Table 2:** Results of synthesis of compound **5** catalyzed by supported Cu(I) and free CuI.

Catalyst	t (h)	%Yield
**supported Cu(I) ^a^**	2	59.9
**CuI ^a^**	75	28.7

^a^ Detailed reaction conditions are reported in the experimental section.

## Supplementary Materials

The following are available online. Table S1: Typical physical & chemical characteristics of Purolite® C104 Plus.; Attachement S2: IR spectra of catalyst in different stages of preparation. Attachement S3: Study of Catalyst Recycling for One-pot Synthesis of 4,4´-[(6-{[4-(Aminosulfonyl)phenyl]amino}-1,3,5-triazine-2,4-diyl)bis(iminoethane-2,1-diyl)dibenzene-sulfonamide (entry 11 in the article).
