# Supplementary material for: New Approach for the One-Pot Synthesis of 1,3,5-Triazine Derivatives: Application of Cu(I) Supported on a Weakly Acidic Cation-Exchanger Resin in a Comparative Study
Source: Molecules. 2019 Oct 5;24(19):3586. doi: 10.3390/molecules24193586 (PMC6804075; doi:10.3390/molecules24193586)
Supplement: Supplementary file 1 [file molecules-24-03586-s001.pdf]

1. Typical Physical & Chemical Characteristics of Purolite® C104 Plus

Table S1. Typical physical & chemical characteristics of Purolite® C104 Plus. [1]

Polymer Structure	Porous crosslinked polyacrylic acid
Appearance	Spherical Beads
Functional Group	Carboxylic Acid
Ionic Form	H^+ form
Total Capacity	4.7 eq/L (102.7 Kgr/ft^3) (H^+ form)
Moisture Retention	45 - 55 % (H^+ form)
Particle Size Range	300 - 1600 µm
< 300 µm (max.)	1 %
Reversible Swelling, $H^+ \rightarrow Ca^{2+}$ (max.)	20 %
Reversible Swelling, $H^+ \rightarrow Ca^{2+}$ (operating)	7% (approximately)
Reversible Swelling, $H^+ \rightarrow Na^+$ (max.)	60 %
Specific Gravity	1.19
Shipping Weight (approx.)	740 - 780 g/L (46.2 - 48.8 lb/ft^3)
Temperature Limit	120 °C (248.0 °F)

2. IR spectra of catalyst in different stages of preparation

The IR spectra with the scheme is reproduced from the Thesis of our colleague Nitin Kore, PhD. [2]

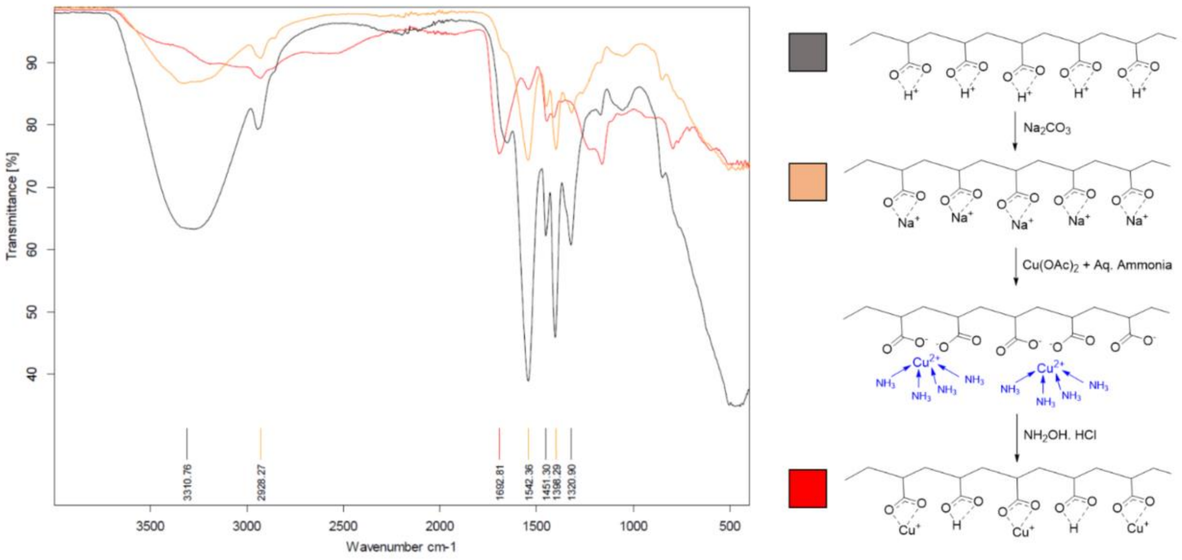

3. Study of Catalyst Recycling for One-pot Synthesis of 4,4′-[(6-{[4-(Aminosulfonyl)phenyl]amino}-1,3,5-triazine-2,4-diyl)bis(iminoethane-2,1-diyl)dibenzene-sulfonamide (entry 11 in the article)

Starting dichlorotriazinyl benzenesulfonamide (1 mmol, 0.320 g) was dissolved in DMF (20 mL). Then solid anhydrous potassium carbonate (0.138 g, 1 mmol) was added in small portions and the mixture was stirred for 10 minutes. Then 4-(2-aminoethyl)benzenesulfonamide (0.200 g, 1 mmol) was added portion wise. Finally, supported Cu(I) catalyst (312 mg, 2.5 % mol.) was added into the reaction

mixture. Reaction was stirred at 35 °C until the maximum conversion of starting material is achieved (monitored by TLC). After completion of the first reaction step, 4-(2-aminoethyl)benzenesulfonamide (0.200 g, 1 mmol) and anhydrous potassium carbonate (0.138 g, 1 mmol) were added into the reaction mixture. The reaction mixture was then stirred at 100 °C until the maximum conversion of a nucleophile is determined (monitored by TLC). After completion of a reaction, the catalyst and salt were filtered off. Crushed ice was then added into the solution and the formed precipitate was collected by filtration. The crude product was dissolved in hot acetone and precipitated by the addition of isopropyl alcohol.

Because of large size of resin beads, all catalyst was recovered very easily by simple filtration. Catalyst recovered from the reaction mixture was at first washed with distilled water to remove the base. Then the catalyst was washed with methanol and dried at room temperature and used for the next run. The same reaction procedure was then repeated several times using the same batch of catalyst each time recycled as described above.

Table S2. Study of recycled catalyst.

**Run No.**	**1**	**2**	**3**	**4**	**5**	**6**	**7**	**8**	**9**	**10**
**Yield (%)**	75	75	73	74	75	72	75	73	73	74
**Time (h)**	8	8	8	8	8	8	8	8	8	8

References

1. Purolite C104 Plus https://www.purolite.com/product/c104plus (accessed Jun.16, 2019).

2. KORE, Nitin Shivanna. *Application Studies of New Stable Inexpensive Ligand-Free Cu(I) Catalyst Supported on Weakly Acidic Polyacrylate Resin in Syntheses*. Brno, 2019. Thesis. Masaryk University, Faculty of Science. Supervisor: Pavel Pazdera. Available from: <https://is.muni.cz/th/dy62d/>.
